# Smoking is associated with higher short-term risk of revision and mortality following primary hip or knee arthroplasty: a cohort study of 272,640 patients from the Dutch Arthroplasty Registry

**DOI:** 10.2340/17453674.2024.39966

**Published:** 2024-02-12

**Authors:** Joris BONGERS, Maartje BELT, Anneke SPEKENBRINK-SPOOREN, Katrijn SMULDERS, B Willem SCHREURS, Sander KOETER

**Affiliations:** 1 Department of Orthopaedic Surgery, Radboud Institute for Health Sciences, Radboud University Medical Centre, Nijmegen; 2 Department of Research and Innovation, Sint Maartenskliniek, Nijmegen; 3 Dutch Arthroplasty Register (Landelijke Registratie Orthopedische Interventies), ‘s-Hertogenbosch; 4 Department of Orthopaedic Surgery, Sint Maartenskliniek, Nijmegen; 5 Department of Orthopaedic Surgery, Canisius Wilhelmina Ziekenhuis, Nijmegen, The Netherlands

## Abstract

**Background and purpose:**

Patients actively smoking at the time of primary hip or knee arthroplasty are at increased risk of direct perioperative complications. We investigated the association between smoking status and risk of revision and mortality within 2 years following hip or knee arthroplasty.

**Methods:**

We used prospectively collected data from the Dutch Arthroplasty Register. All primary total hip arthroplasties (THAs), total knee arthroplasties (TKAs), and unicondylar knee arthroplasties (UKAs) with > 2 years’ follow-up were included (THA: n = 140,336; TKA: n = 117,497; UKA: n = 14,807). We performed multivariable Cox regression analyses to calculate hazard risks for differences between smokers and non-smokers, while adjusting for confounders (aHR).

**Results:**

The smoking group had higher risk of revision (THA: aHR 1.3, 95% confidence interval [CI] 1.1–1.4 and TKA: aHR 1.4, CI 1.3–1.6) and risk of mortality (THA: aHR 1.4, CI 1.3–1.6 and TKA: aHR 1.4, CI 1.2–1.6). Following UKA, smokers had a higher risk of mortality (aHR 1.7, CI 1.0–2.8), but no differences in risk of revision were observed. The smoking group had a higher risk of revision for infection following TKA (aHR 1.3, CI 1.0–1.6), but not following THA (aHR 1.0, CI 0.8–1.2).

**Conclusion:**

This study showed that the risk of revision and mortality is higher for smokers than for non-smokers in the first 2 years following THA and TKA. Smoking could contribute to complications following primary hip or knee arthroplasty.

Primary hip and knee arthroplasty constitutes a large role in the Dutch healthcare system with more than 60,000 operations every year [1]. Smoking is one of the modifiable patient-related factors leading to surgical complications, such as delayed wound healing and surgical site infection [2]. Although the prevalence of smoking is decreasing worldwide, the expected absolute number of smoking patients is still likely to grow in the near future due to increasing demand for total hip (THA) and total knee arthroplasty (TKA) [3,4].

Smoking can be a predictor of poor outcome and previous studies have reported smoking as a risk factor for periprosthetic joint infection (PJI), aseptic loosening, and revision [5-8]. Smaller registry studies showed conflicting results regarding the effects of smoking on revision rates [9,10]. A database study showed that for unicondylar knee arthroplasty (UKA), smokers are at higher risk for any wound complication and reoperation, but this study did not specifically address revision and mortality rates [11]. Our study is the first to investigate a possible association between smoking and outcome following UKA and the largest register study to investigate the association following primary hip and knee arthroplasty.

We aimed to investigate the association between smoking status and the outcome of primary hip and knee arthroplasty in a large cohort derived from the Dutch Arthroplasty Register (LROI). The primary outcome measures were defined as risk of revision and mortality. We expected the risk of revision and mortality to be higher in smokers, with higher risk of revision due to higher rates of PJIs. Therefore, we also investigated the association between smoking and revision due to infection.

## Patients and methods

### Dutch Arthroplasty Register (LROI)

We performed an observational study using LROI data. The LROI is a database containing prospectively collected data reported by all hospitals performing arthroplasties in the Netherlands. The completeness has been more than 98% for primary arthroplasty and more than 97% for revision arthroplasty from 2015 onward [1]. All patients have a unique identification number that connects demographic data, the primary and possible subsequent revision arthroplasty. Patients can choose to opt out of the LROI register. The LROI contains demographic information, surgical variables, and prosthesis characteristics of all primary and revision arthroplasty procedures. Smoking behavior, Charnley score, body mass index (BMI), and patient-reported outcome measures (PROMs) have been registered since 2014. All demographic information is registered in the preanesthetic evaluation. Smoking behavior is categorized as smoker or non-smoker, based on self-reported smoking status. No information is available on smoking history. A revision is defined as any change (insertion, replacement, and/or removal) of one or more components of the prosthesis. We defined short-term revision and mortality as revision or death within 2 years following the index operation. Reasons for revision are categorized by the surgeon at the time of revision surgery and more than one reason for revision can be registered. In the event of death, the date of death is added to the database based on the national insurance database.

### Data selection

All THAs, TKAs, and UKAs performed for any diagnosis since January 1, 2014 with a 2-year follow-up until January 1, 2021 and a known smoking status were included. Patients with missing information on smoking history were excluded. Patients with bilateral arthroplasty were included. All diagnoses prior to surgery were included. Invalid data on age (> 105 years and < 10 years) and BMI (> 70 and < 10) were adjusted to missing data. Patients with the combination age < 18 years and the diagnosis osteoarthritis were also excluded. Patients were divided into subgroups according to type of surgery (THA, TKA, and UKA), because of the differences in soft-tissue handling and the effect of smoking behavior on the healing of soft tissue [2].

### Statistics

**Figure 1:**
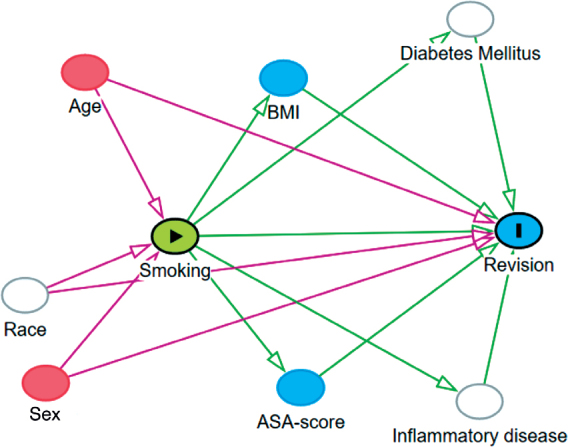
A directed acyclic graph (DAG) to test which confounding factors should be accounted for. In all analyses, age and sex were identified as confounding factors and adjusted for. Ethnicity, diabetes mellitus, and inflammatory diseases were also determined as possible confounding factors for revision but, these are not reported to the LROI and therefore could not be adjusted for.

Kaplan–Meier curves were generated to demonstrate the survival probability of each subgroup. To evaluate differences in short-term risk of revision and mortality between smokers and non-smokers, multivariable Cox regression analyses were run returning crude hazard ratios (HR) and adjusted hazard ratios (aHR) for each subgroup, while adjusting for the confounding factors age and sex. Post-hoc analyses were performed for each subgroup to study differences in short-term risk of revision due to infection and all other reasons for revision between smokers and non-smokers. Results are presented as HR with 95% confidence interval (CI). A directed acyclic graph (DAG) was made for each subgroup, to test which confounding factors should be accounted for in our models (Figure 1 see Appendix). For each subgroup in all analyses, age and sex were identified as confounding factors and adjusted for. Ethnicity, diabetes mellitus (DM), and inflammatory diseases were also determined as possible confounding factors for revision. However, these are not reported to the LROI and therefore could not be adjusted for.

Unmeasured confounders, such as myocardial infarction and cerebrovascular accidents, are even more numerous in the analysis of the association between smoking and mortality. To explore the possible effect of these unmeasured confounders, we did a sensitivity analysis. We calculated the E-value to quantify the minimum strength of association that unmeasured confounding factors would need to have to fully explain away a specific treatment–outcome association [12]. We could not perform a sensitivity analysis including the data of excluded patients with no information on smoking history. All our analyses were performed assuming that the data from patients with no information on smoking history was missing completely at random (MCAR) [13].

No further analyses on PROMs were performed due to the large percentage of missing data in each subgroup.

All analyses were performed using packages haven, table 1, survival and E-Value of R version 4.1.1 RStudio Team (2020). RStudio: Integrated Development for R. Boston (USA) [14]. The study was conducted and reported according to STROBE guidelines.

**Table 1:** Patient demographics and results of THA, TKA, and UKA group. Values are count (%) unless otherwise specified

Factor	THA		TKA		UKA	
	Smokers (n = 16,299)	Non-smokers (n = 124,037)	Smokers (n = 10,614)	Non-smokers (n = 106,883)	Smokers (n = 1,629)	Non-smokers (n = 13,178)
Sex						
female	9,378 (60)	81,897 (66)	5,625 (53)	69,428 (65)	849 (52)	7,446 (56)
male	6,543 (40)	42,056 (34)	4,982 (47)	37,376 (35)	779 (48)	5,725 (44)
missing	18 (0)	87 (0)	7 (0)	79 (0)	1 (0)	5 (0)
Age, mean (SD)	63.7 (10.8)	69.6 (10.3)	63.9 (9.1)	69.0 (9.0)	59.9 (8.0)	63.8 (8.7)
range	16–103	13–103	17–92	12–98	34–88	32–101
missing	13 (0)	76 (0)	5 (0)	64 (0)	1 (0)	7 (0)
ASA score						
1	2,578 (16)	22,244 (18)	1,420 (14)	14,222 (13)	349 (21)	2,997 (23)
2	10,747 (66)	79,711 (64)	7,225 (68)	72,800 (68)	1,126 (69)	8,653 (66)
3–4	2,940 (18)	21,954 (18)	1,956 (18)	19,747 (19)	153 (10)	1,519 (11)
missing	34 (0)	128 (0)	13 (0)	114 (0)	1 (0)	9 (0)
BMI, mean (SD)	26.7 (4.8)	27.4 (4.5)	29.3 (5.1)	29.8 (5.0)	28.9 (4.4)	29.3 (4.5)
range	14–58	10–70	11–57	11–68	11–51	14–69
missing	69 (0.4)	779 (0.6)	33 (0.3)	505 (0.5)	8 (1)	69 (1)
Revisions **^a^**	471 (2.9)	2,872 (2.3)	377 (3.6)	2,733 (2.6)	76 (4.6)	525 (3.9)
Mortality **^a^**	629 (3.9)	3,322 (2.7)	250 (2.4)	1,842 (1.7)	18 (1.1)	112 (0.8)

a Revision and mortality numbers are events before 2 years of follow-up.

SD = standard deviation; THA = total hip arthroplasty; TKA = total knee arthroplasty; UKA = unicondylar knee arthroplasty; ASA = American Society of Anesthesiologists; BMI = body mass index.

### Ethics, funding, data sharing, and disclosures

Ethical approval for the current study was not applicable according to the Dutch Medical Research Involving Human Subjects Act. No funding was received. Data was made available by the LROI; however, restrictions apply to the availability of this data, which was used under license for the current study. There are no conflicts of interest. Complete disclosure of interest forms according to ICMJE are available on the article page, doi: 10.2340/17453674.2024.39966

## Results

All primary arthroplasties of the hip and knee in the LROI since January 1, 2014 were included (n = 404,389). After exclusion of patients with missing information on smoking history, patients were divided in subgroups according to type of surgery and categorized as smokers or non-smokers.

**Figure 2:**
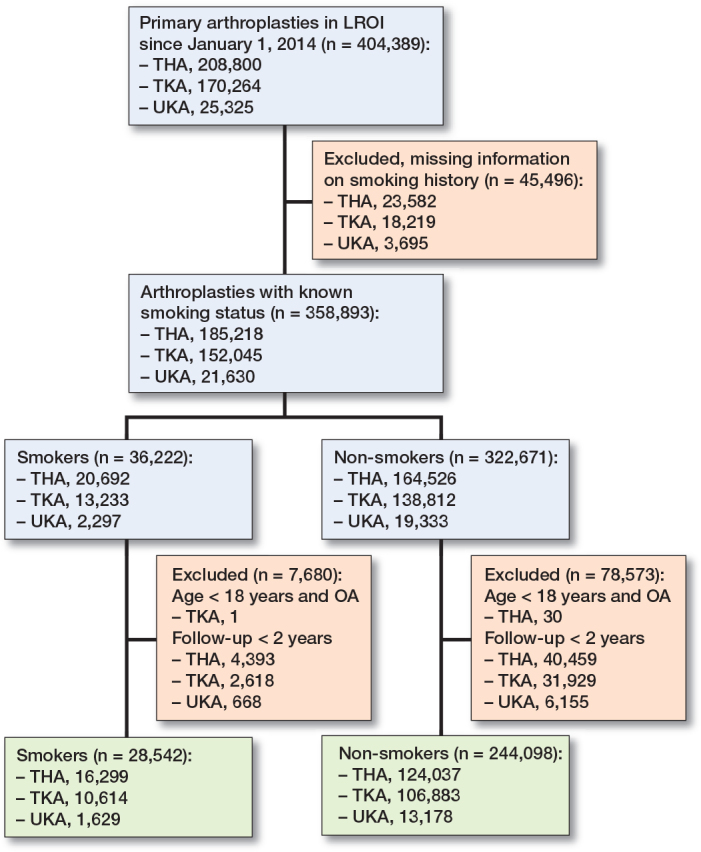
Flowchart of data selection. LROI = Landelijke Registratie Orthopedische Interventies; THA = total hip arthroplasty; TKA = total knee arthroplasty; UKA = unicondylar knee arthroplasty.

**Figure 3:**
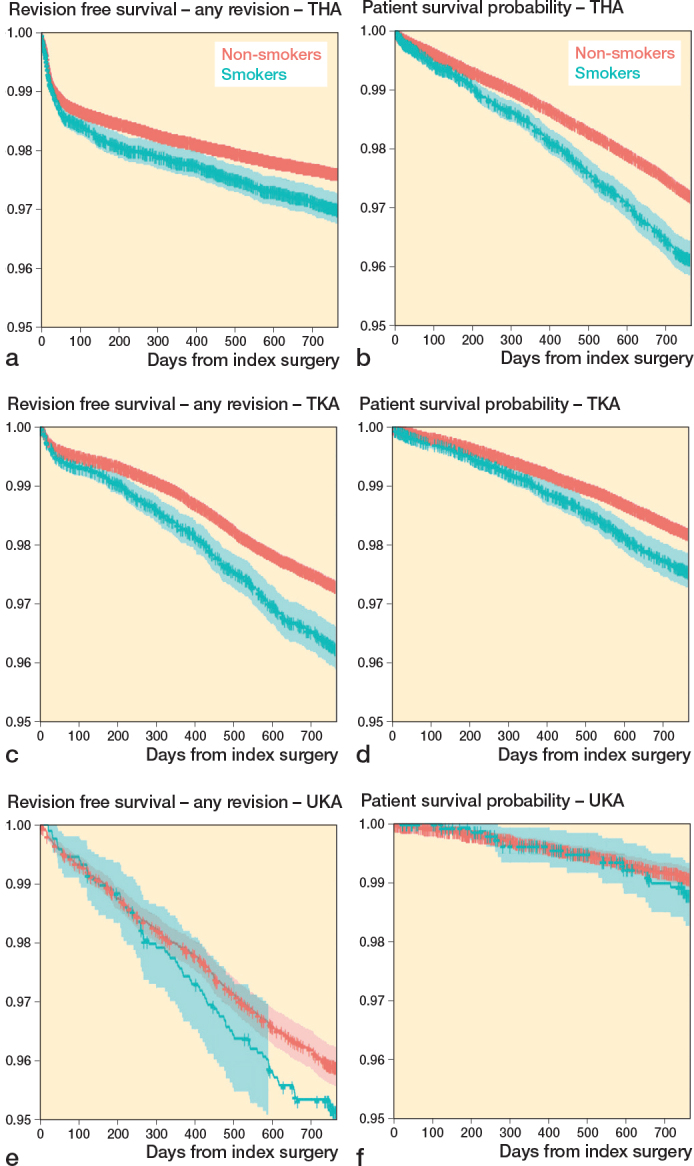
Kaplan–Meier implant survival probability with revision for any reason as the endpoint following (a) total hip arthroplasty (THA),(c) total knee arthroplasty (TKA), and (e) unikompartmental knee arthroplasty (UKA). Kaplan–Meier patient survival probability with death as the endpoint following (b) THA, (d) TKA, and (f) UKA.

272,640 patients with follow-up > 2 years were included in the study (140,336 THAs, 117,497 TKAs, and 14,807 UKAs) (Figure 2). The percentage of smokers differed among the subgroups (9–12%). Men are more likely to be smokers and smokers tend to be younger than non-smokers. BMI and ASA score were comparable in all subgroups (Table 1). Infection and instability were the most common reason for revision, followed by aseptic loosening (Table 2). The Kaplan–Meier analyses indicate a worse survival prognosis for smokers for revision and mortality in THA and TKA (Figure 3a–d). For UKA, a higher event rate is seen for revision but not for mortality (Figure 3e–f).

**Table 2:** Absolute numbers and percentages of reasons for revision following THA, TKA, and UKA

Reason	THA		TKA		UKA	
	Smokers (n = 16,299)	Non-smokers (n = 124,037)	Smokers (n = 10,614)	Non-smokers (n = 106,883)	Smokers (n = 1,629)	Non-smokers (n = 13,178)
Infection	126 (0.8)	920 (0.7)	93 (0.9)	690 (0.6)	6 (0.4)	46 (0.3)
Instability	151 (0.9)	751 (0.6)	114 (1.1)	665 (0.6)	20 (1.2)	100 (0.8)
Aseptic loosening	98 (0.5)	623 (0.5)	51 (0.5)	484 (0.4)	18 (1.1)	118 (0.9)
Periprosthetic fracture	83 (0.5)	460 (0.3)	9 (0.0)	65 (0.0)	3 (0.2)	25 (0.2)
Wear	8 (0.0)	38 (0.0)	8 (0.0)	31 (0.0)	1 (0.1)	16 (0.1)
Patellar disorders			114 (1.1)	773 (0.7)	7 (0.4)	25 (0.2)
Arthrofibrosis			29 (0.3)	235 (0.2)	1 (0.1)	9 (0.1)
Malposition			40 (0.4)	336 (0.3)		
Progression of OA				15 (0.9)	87 (0.7)	
Miscellaneous	68	441	38	292	14	159
Conversion to TKA					41 (2.5)	287 (2.2)
Revision UKA					29 (1.8)	190 (1.4)

For Abbreviations, see Table 1, and OA = osteoarthritis.

### Risk of revision within 2 years (Table 3)

For THA, there were 471 revisions (2.9%) in the smoking group and 2,872 revisions (2.3%) in the non-smoking group. A higher risk of revision was observed in the smoking group compared with non-smokers (HR 1.3, CI 1.1–1.4 and aHR 1.2, CI 1.1–1.4).

**Table 3:** Adjusted (aHR) and crude hazard ratios (HR) with 95% confidence interval (CI) for comparison of revision and mortality rates between smokers and non-smokers

	Risk of revision within 2 years		Risk of mortality within 2 years	
	aHR (CI)	HR (CI)	aHR (CI)	HR (CI)
THA	1.2 (1.1–1.4)	1.3 (1.1–1.4)	2.3 (2.1–2.5)	1.5 (1.3–1.6)
TKA	1.2 (1.1–1.4)	1.4 (1.3–1.6)	2.1 (1.8–2.4)	1.4 (1.2–1.6)
UKA	1.1 (0.8–1.3)	1.2 (0.5–2.3)	1.7 (1.0–2.8)	1.3 (0.8–2.2)

aHR = hazard ratio adjusted for age and sex.

For Abbreviations, see Table 1.

For TKA, a similar pattern was observed. Absolute differences remained small: revision risk was 3.6% in the smoking group and 2.6% in the non-smoking group. This resulted in a higher risk of revision with an HR of 1.4 (CI 1.3–1.6), and an aHR of 1.2 (CI 1.1–1.4).

For UKA no differences were seen. There were 76 revisions (4.6%) in the smoking group and 525 revisions (3.9%) in the non-smoking group with HR 1.2 (CI 0.9–1.5).

Post-hoc analysis showed that, for THA, smokers have a higher 2-year risk of revision for instability (aHR 1.6, CI 1.4–1.9) and periprosthetic fracture (aHR 1.5, CI 1.2–2.0) compared with non-smokers, but not for infection (aHR 1.0, CI 0.8–1.2). For TKA, smokers had a higher risk of revision for infection (aHR 1.3, CI 1.0–1.6), instability (aHR 1.4, CI 1.1–1.7), and patellar disorders (aHR 1.3, CI 1.1–1.6) than non-smokers. HR was adjusted for age and sex. In patients undergoing UKA, no statistically significant differences were found in 2-year revision risk for specific reasons between smokers and non-smokers.

### Mortality within 2 years

For THA, a higher mortality risk was observed in the smoking group (HR 1.5, CI 1.3–1.6 and aHR 2.3, CI 2.1–2.5) corresponding to 3.9% in the smoking group and 2.7% in the non-smoking group (Table 2). The absolute number was 599 deaths.

The same was observed for mortality following TKA, with 2.4% dead in the smoking group, compared with 1.7% in the non-smoking group, resulting in an HR of 1.4 (CI 1.2–1.6) and an aHR of 2.1 (CI 1.8–2.4).

Following UKA, mortality rates were lower than after a THA or TKA; however, for smokers they were also slightly higher than for non-smokers: 1.1% versus 0.8% (HR 1.3, CI 0.8–2.2 and aHR 1.7, CI 1.0 – 2.8).

### Effect of unmeasured confounders

The calculated E-values for revision for each subgroup were: 1.8 for THA, 1.8 for TKA, and 1.3 for UKA. For mortality, the E-values were: 3.9 for THA, 3.6 for TKA, and 2.8 for UKA.

## Discussion

We performed the largest study to date aiming to compare the short-term risks of revision and mortality of smoking and non-smoking patients receiving a THA or TKA and to analyze associations between smoking and risk of revision in UKA. The most important finding of this study is that smoking was associated with a higher risk of revision and mortality following THA and TKA. Following UKA, we found only a weak association between smoking and short-term mortality.

Our findings may be explained by multiple pathophysiological pathways toward failure of a prosthesis in which smoking plays a role. Sorensen described the pathophysiological effect smoking has on surgical wounds [2]. Smoking increases oxidative stress, leading to physiologic responses, inflammatory responses, and proliferative responses obstructing tissue healing. This could lead to complications such as cell necrosis, wound dehiscence, surgical site and deep infection, delayed tissue and bone healing, and increased risk of PJIs, which we expected. We found differences in revisions due to an infection between smokers and non-smokers following TKA, but not for THA or UKA (see Table 2).

The negative impact of nicotine and cigarette smoke on the physiological pathway of osseointegration possibly increases the risk of aseptic loosening [15,16]. In contrast to our study, where we found a higher risk of revision due to infection only following TKA, higher rates of deep infections and aseptic loosening have been reported in smokers following primary hip and knee arthroplasty [6-8,17-20].

Our data does not imply that smokers should not be accepted for arthroplasty surgery on the basis of their smoking behavior. In our study, the association of the isolated risk factor smoking with revision is weak. Adjusted HRs for THA and TKA are low and our sensitivity analysis shows that unmeasured confounders could contribute to this effect. Furthermore, the absolute revision and mortality rates are low. The data is supported by previous literature with low absolute numbers of revision and mortality in all studies [5,9,10,19,21]. A meta-analysis of 4 studies showed that smokers had an increased risk of revision with a risk ratio of 2.6 (CI 1.3–5.2) [6]. Smaller recent register studies even fail to demonstrate higher revision rates for smokers following primary hip and knee arthroplasty, but these may be underpowered [9,10]. Follow-up in the study by Matharu et al. was up to 20 years, possibly clouding the early effect smoking behavior could have on revision rates [9]. To patients, function, pain status, and quality of life, as expressed in PROMS, are of more importance than revision percentages following primary hip and knee arthroplasty [22]. Smokers do benefit equally from arthroplasty surgery compared with non-smokers [9,23]. However, smokers should be informed about the associated revision and mortality risks during the shared decision-making process. Smoking cessation prior to surgery might partly reverse the detrimental effects of smoking on tissue healing [2]. Intensive smoking cessation programs reduce the occurrence of complications and can lead to prolonged smoking cessation following surgery [24-26]. Although no literature exists on smoking cessation and its association with revision and mortality rates following primary hip and knee arthroplasty, such programs can be encouraged in the weeks leading up to surgery for general health purposes. Surgery has been proven to be an extra motivation to quit smoking and smoking cessation programs have a large beneficial health effect [27]. Several studies have shown the cost-effectiveness of smoking cessation programs prior to surgery [28,29].

Our data also indicate that the risk of mortality during the first 2 years following THA and TKA is more than twice as high in smokers compared with non-smokers. These findings resemble the results described in previous literature [9]. Our HRs are similar to the register study performed by Matharu et al. for both THA and TKA [9].

It is possible that unmeasured confounders constitute some effect in the calculated association of smoking. The E-values are higher than the adjusted HRs for the different subgroups, indicating that (a set of) unmeasured confounding factors need to have a stronger association to negate the association between smoking and revision. However, because of the low adjusted HRs in revision the association needed is small. Therefore, it is likely that the unmeasured confounding factors explain some of the association between smoking and revision. Our analyses are limited to the risk factors that are registered by the LROI. It is possible that revision rates will change if we can integrate the unmeasured confounding factors race and DM into our analysis. Of these risk factors we value DM as the single most important unmeasured variable. In a systematic review by Jasper et al., other studies mentioned higher associations between these risk factors and revision following TKA than we found for smoking [30].

Similar results in risk of revision were found for smokers and non-smokers following UKA. The overall risk of revision is higher following UKA than after THA or TKA, but this is due to a trend to convert patients with an unsatisfactory outcome after a UKA to a TKA. The mortality after a UKA is lower than after a THA or TKA; the reason may be patient selection as patients with an UKA are overall younger. However, smokers did have only a slightly higher risk of death following UKA, but with a smaller HR than the THA and TKA patients. UKA is performed less frequently than THA or TKA and, possibly, a larger number of patients is needed to yield the effect of smoking in this subgroup. Another possible explanation is that UKA has a less invasive character and allows for earlier mobilization and rehabilitation [31]. So far, only 1 study has investigated the effects of smoking on UKA outcome [11]. This study showed a higher risk for early wound complications and reoperations but did not demonstrate an association with PJIs [11].

### Strengths and limitations

A major strength of this register study is that the LROI is a population-based registry with more than 95% completeness. The proportion of missing data was very low; thus we do not expect that this missing data influences our findings.

The first limitation is that arthroplasty registry data are observational data, therefore residual confounding may remain despite our efforts to minimize this effect. Residual confounding could lead to an overestimation of the association of smoking with the postoperative outcomes. Second, although mortality is registered in the LROI registry, cause of death is unknown, and we cannot conclude deaths are directly related to smoking behavior. Third and most importantly, categorizing smoking status to smoking and non-smoking may lead to underestimation of the effect of smoking on postoperative outcomes. No information was available on former smoking, exposure to smoking (passive smoking, cigarettes per day, tobacco content) or history of smoking (pack years). It was not possible to analyze any relationship between exposure and arthroplasty outcome. Smoking is categorized based on patient self-report. Selfreported non-smokers may have concealed their smoking behavior because of social or medical disapproval. This could have led to an underestimation of smoking patients. However, both poor and good outcomes of these smokers are listed under the non-smoking group. Therefore, we do not expect this underestimation to alter the results. It is possible that former smoking increases the complication rate in the non-smokers group, since some of the detrimental pathophysiological effects of smoking are irreversible and might have occurred before smoking cessation [2]. Fourth, outcome measures can be underreported in registry data. Infections are known to be underreported in the LROI register [32]. This recent study found that up to 53% of acute PJIs were not reported in the LROI register. In half of the cases, the acute PJI was treated with a reoperation without exchange of one of the components and therefore not registered as a revision. In the other half, the missing cases were of administrative error. Under-registration of PJIs could have led to incorrect revision rates for smokers and non-smokers. Although there is no reason to suspect the underestimation to differ between the 2 groups, it is possible revision rates due to infections are, in reality, higher. Fifth, patients with no information on smoking behavior were excluded from this study. We have assumed the missing information on smoking status to be MCAR [13]. These patients accounted for 11% of all the patients in the LROI registry and their missing data could potentially influence our results if they prove not to be MCAR. Ideally, the assumed MCAR of smoking status should have been tested using a sensitivity analysis in which we imputed smoking status. This was not possible because patients with missing information on smoking status were excluded by the LROI earlier in the study process. Finally, our findings were derived from the Dutch arthroplasty register and may not apply to other populations with different patient characteristics or from different healthcare systems.

### Conclusion

We found higher risks of short-term revision and mortality for smokers than for non-smokers in the first 2 years following primary hip and knee arthroplasty. The isolated risk factor smoking contributes to a higher change in short-term revision and mortality following primary hip or knee arthroplasty.

In perspective, these findings do not implicate that smokers should be denied primary hip and knee arthroplasty but smokers should be informed of the associated risks of revision and mortality.

## Notes

JB conceived and designed the analysis, collected the data, contributed data or analysis tools, performed the analysis, and wrote the paper. MB conceived and designed the analysis, performed the analysis, and revised the paper. AS collected the data, contributed data or analysis tools, and revised the paper. KS, BWS, and SK conceived and designed the analysis, and revised the paper.

Handling co-editors: Marianne Westberg and Robin Christensen

*Acta* thanks Håvard Dale and Kaisa Huotari for help with peer review of this manuscript.
